# A Case of Superficial True Venous Aneurysm of the Upper Extremity

**DOI:** 10.3400/avd.cr.26-00040

**Published:** 2026-07-23

**Authors:** Hiroki Nakabori, Toshiko Kakiuchi, Kenji Iino

**Affiliations:** 1 Department of Cardiovascular Surgery, Municipal Tsuruga Hospital, Tsuruga, Fukui, Japan; 2 Department of Pathology, Municipal Tsuruga Hospital, Tsuruga, Fukui, Japan; 3 Department of Cardiovascular Surgery, Kanazawa University, Kanazawa, Kanazawa, Japan

**Keywords:** venous aneurysm, upper extremity, true aneurysm

## Abstract

Venous aneurysms of the upper extremity have rarely been reported. A 78-year-old woman presented with swelling, discomfort, and mild tenderness on the medial side of the right elbow. Ultrasonography showed a 2.1 × 1.8 cm dilated structure connected to the basilic vein. Because a venous pseudoaneurysm was suspected and the patient had discomfort and mild tenderness, aneurysm excision and venous reconstruction were performed under local anesthesia. Histopathological examination confirmed a true venous aneurysm. The postoperative course was uneventful, with complete symptom resolution and no recurrence at 6 months.

## Introduction

A venous aneurysm is a localized dilatation of a vein without associated elongation or tortuosity and is distinct from a varicose vein.^1)^ It is a relatively rare disease. Reports of venous aneurysms of the upper extremities are particularly uncommon, whereas popliteal venous aneurysms are more frequently reported among extremity lesions.^2)^ Here, we report a case of a true venous aneurysm of the basilic vein in the upper arm, a condition that has been reported only infrequently in the literature.^3)^

## Case Report

**Fig. 1:**
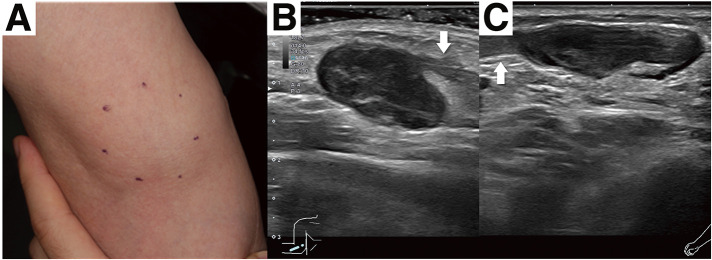
Preoperative photograph and ultrasonographic findings of the right elbow. (**A**) A mass lesion measuring approximately 3 cm in diameter was present on the medial side of the right elbow. The lesion was soft to slightly firm and associated with mild tenderness. (**B**) Ultrasonography showed a dilated structure measuring 2.1 × 1.8 cm connected to the basilic vein, with no internal blood flow signal on the short-axis view. The white arrow denotes the proximal basilic vein. (**C**) Long-axis ultrasonographic view of the lesion. The white arrow denotes the distal basilic vein.

**Fig. 2:**
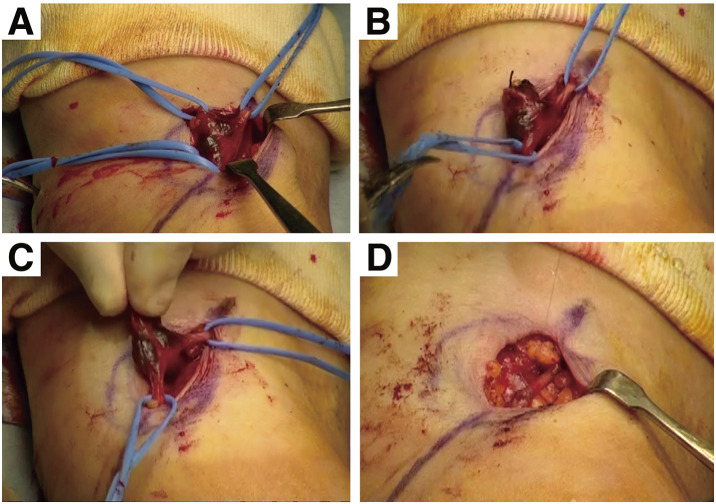
Intraoperative findings. (**A**) Intraoperative photograph. The caudal side is shown at the bottom. The tissue surrounding the aneurysm was dissected, and the inflow vessel, outflow vessel, and small branches were secured. (**B**) Intraoperative photograph after ligation and division of the small branches. The inflow vessel is shown at the bottom and the outflow vessel at the top. (**C**) Dilatation of the main trunk of the basilic vein was observed, along with dark discoloration suggestive of the presence of an intraluminal thrombus. (**D**) Intraoperative photograph after aneurysm resection and end-to-end venous anastomosis.

**Fig. 3:**
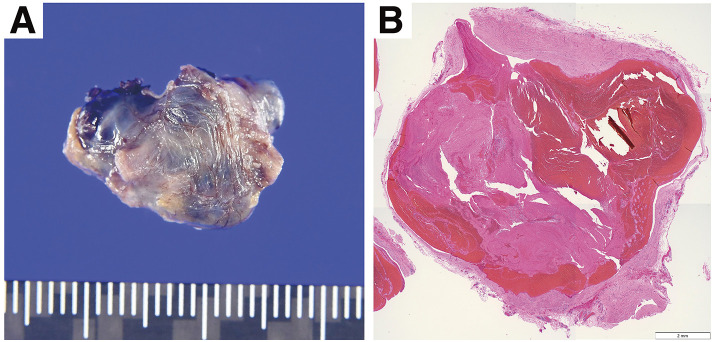
Resected specimen and histopathological findings. (**A**) Resected specimen measuring 1.9 × 1.5 cm. (**B**) Histopathological specimen showing that the outermost layer of the aneurysm consisted of a vascular wall containing a muscular layer and that the lumen contained partially thrombosed blood, consistent with a true aneurysm.

A 78-year-old woman presented with swelling and discomfort around the right elbow. Approximately 5–6 years earlier, she had experienced swelling at the same site and underwent needle aspiration at a local clinic, yielding blood-like fluid, after which she was followed conservatively with compression. Although the swelling subsided, she noticed a recurrent subcutaneous nodule on the medial side of the right elbow approximately 1 month before presentation and visited the dermatology department at our hospital (**Fig. 1A**). Ultrasonography showed an aneurysmal venous lesion with thrombus, and she was referred to our department. She had no history of trauma. Her medical history included an anterior communicating artery aneurysm and overactive bladder. She had no history of surgery. Her only medication was for overactive bladder. She denied repetitive irritation or manipulation of the lesion. On physical examination, a soft to slightly firm mass measuring approximately 3 cm in diameter was palpable on the medial side of the right elbow. Mild tenderness was present. Ultrasonography revealed a mass lesion measuring approximately 2.1 × 1.8 cm arising from the main trunk of the basilic vein in the upper arm. Most of the lesion showed no color Doppler signal, and sludge echo was observed within the mass, whereas a slight signal was detected at the portion considered to be connected to the vessel. The lesion was easily deformable on compression testing. Its margin was slightly irregular, and in some areas the border with the surrounding tissue was indistinct (**Fig. 1B** and **1C**). Given the history of previous needle aspiration, a venous pseudoaneurysm was suspected. Because the patient also had discomfort and mild tenderness at the lesion site and wished to undergo surgery, surgical treatment was planned. A vertical skin incision approximately 4 cm in length was made directly over the lesion. The inflow and outflow vessels of the aneurysm were identified and secured. After sufficient dissection of these vessels, the aneurysm was resected. The venous stump was reconstructed by continuous suturing of approximately 2-thirds of the posterior wall using 7-0 polypropylene with a 1-point support technique, followed by end-to-end anastomosis of the anterior wall with 4 interrupted 7-0 polypropylene sutures (**Fig. 2**). Doppler examination confirmed satisfactory blood flow after reconstruction. Histopathological examination revealed that the outermost layer of the lesion consisted of a vascular wall containing a muscular layer, with partially thrombosed blood within the lumen. Part of the thrombus showed organization, with proliferation of fibrous connective tissue and integration into the vascular wall. Focal thinning of the vascular wall was observed, and in a very limited area, the wall had ruptured and was directly in contact with the surrounding adipose tissue; however, the lesion as a whole was diagnosed as a true venous aneurysm (**Fig. 3**). After surgery, the patient's discomfort and tenderness resolved completely, and her postoperative course has been uneventful. No recurrence has been observed during approximately 6 months of follow-up.

## Discussion

A true venous aneurysm is defined as an isolated venous dilatation with preservation of the normal 3-layered venous wall structure. In addition, it is not associated with arteriovenous communication, pseudoaneurysm, or varicose vein.^1)^ Venous aneurysms of the upper extremity are estimated to account for 4.2% of all venous aneurysms,^4)^ and they can be classified as deep or superficial according to their location.^5)^ Superficial venous aneurysms have been reported to account for approximately 0.1% of venous aneurysms, and those arising in the upper extremity have been reported less frequently, with only a limited number of cases described in the literature.^3)^ Skandali et al. searched English-language articles available in PubMed and Google Scholar up to June 2025 and identified 22 cases of superficial true venous aneurysm.^3)^ However, because these lesions are generally benign and asymptomatic, similar cases encountered in clinical practice may remain untreated and therefore unreported, which may partly explain the limited number of published cases.

The natural history of venous aneurysms remains unclear, and their etiology is still largely idiopathic.^6)^ Venous aneurysms can generally be diagnosed by careful physical examination and ultrasonography; however, thrombosed and occluded lesions may not collapse on palpation and may sometimes be difficult to distinguish from soft-tissue tumors.^7)^ Superficial venous aneurysms are usually asymptomatic and often present as painless, palpable, and easily compressible superficial masses. In contrast, deep venous aneurysms more often present with painful swelling and may be complicated by deep vein thrombosis or pulmonary embolism.^6)^ The differential diagnosis of a venous aneurysm includes benign lipoma, melanocytic lesions, and other vascular lesions.^8)^

Kuntz et al. proposed a diagnostic algorithm for upper-extremity venous aneurysms and emphasized that ultrasonography is the essential first-line modality for evaluating the exact location, intramural thrombus, and feeding vessels.^9)^ Although computed tomography angiography and magnetic resonance angiography may also be useful, they suggested that these modalities are less indispensable than in arterial aneurysms because venous reconstruction is not always necessary in venous lesions other than those involving the deep venous system, such as the axillary or brachial veins.^9)^

Indications for treatment include symptomatic lesions, enlarging lesions, lesions at high risk of thrombosis, uncertain diagnosis, and cosmetic concerns. Saccular aneurysms and large fusiform aneurysms are also considered candidates for intervention.^3)^ Surgical excision is regarded as the first-line treatment because it enables complete removal, provides symptom relief in symptomatic cases, and allows definitive histopathological diagnosis.^3)^ Venous aneurysms in the superficial venous system do not always require reconstruction and may be managed with simple ligation and excision, with no reported post-resection complications.^9)^ On the other hand, sclerotherapy with percutaneous polidocanol injection has also been reported as a simple and minimally invasive option, but caution is needed because procedure-specific complications such as superficial erythema, skin induration, and hyperpigmentation may occur.^6)^

In the present case, surgery was performed because a venous pseudoaneurysm was suspected and the patient had discomfort and mild tenderness at the lesion site, indicating that preoperative differentiation may be challenging in thrombosed lesions. Because the vein could be sufficiently mobilized intraoperatively, complete aneurysm resection followed by end-to-end venous reconstruction was performed. Postoperatively, there was no forearm edema, and the patient's discomfort and pain resolved completely.

## Conclusion

We report a case of superficial true venous aneurysm of the upper extremity, which has rarely been reported. Aneurysm resection with venous reconstruction provided a favorable outcome.

## References

[1] Gillespie DL, Villavicencio JL, Gallagher C, et al. Presentation and management of venous aneurysms. J Vasc Surg 1997; 26: 845–52. 9372824 10.1016/s0741-5214(97)70099-5

[2] Teter KA, Maldonado TM, Adelman MA. A systematic review of venous aneurysms by anatomic location. J Vasc Surg Venous Lymphat Disord 2018; 6: 408–13. 29661366 10.1016/j.jvsv.2017.11.014

[3] Skandali A, Mulita F, Stathopoulou M, et al. Superficial venous aneurysms: a case-based review. Arch Med Sci Atheroscler Dis 2025; 10: 198–210. 10.5114/amsad/209875 PMC12550692 41142694

[4] Faraj W, Selmo F, Hindi M, et al. Cephalic vein aneurysm. Ann Vasc Surg 2007; 21: 804–6. 17980799 10.1016/j.avsg.2007.03.020

[5] Ioannidis O, Varnalidis I, Alexandris K, et al. Double primary venous aneurysm of the small saphenous vein. Ann Ital Chir 2016; 87: S2239253X16026177. 27807320

[6] Mahajan A, Fazal ST, Mehta S, et al. Cephalic vein aneurysm in the distal forearm managed with sclerotherapy-a rare case report and literature review. Int J Angiol 2022; 31: 92–6. 35864889 10.1055/s-0042-1743252 PMC9296263

[7] Murakami M, Seyama A. A case of an upper extremity venous aneurysm. J Jpn Coll Angiol 2022; 62: 31–4. (in Japanese)

[8] McKesey J, Cohen PR. Spontaneous venous aneurysm: report of a non-traumatic superficial venous aneurysm on the distal arm. Cureus 2018; 10: e2641. 30034963 10.7759/cureus.2641 PMC6050165

[9] Kuntz S, Lejay A, Georg Y, et al. Management of upper extremity aneurysms: a systematic review. Int Angiol 2020; 39: 161–70. 32052949 10.23736/S0392-9590.20.04307-2

